# Maternal and perinatal conditions and the risk of developing celiac disease during childhood

**DOI:** 10.1186/s12887-016-0613-y

**Published:** 2016-06-08

**Authors:** Fredinah Namatovu, Cecilia Olsson, Marie Lindkvist, Anna Myléus, Ulf Högberg, Anneli Ivarsson, Olof Sandström

**Affiliations:** Department of Public Health and Clinical Medicine, Epidemiology and Global Health, Umeå University, SE-901 87 Umeå, Sweden; Department of Food and Nutrition, Umeå University, Umeå, Sweden; Department of Women’s and Children’s Health, Obstetrics and Gynaecology, Uppsala University, Uppsala, Sweden; Department of Clinical Sciences, Pediatrics, Umeå University, Umeå, Sweden

**Keywords:** Celiac disease, Caesarean, Children, Delivery, Elective, Income, Infections, Perinatal, Pregnancy, Register

## Abstract

**Background:**

Celiac disease (CD) is increasing worldwide, which might be due to the changing environmental and lifestyle exposures. We aimed to explore how conditions related to maternity, delivery and the neonatal period influence CD onset during childhood.

**Methods:**

Using Sweden’s national registers we had access to information on 1 912 204 children born between 1991 and 2009, 6 596 of whom developed CD before 15 years of age. Logistic regression analyses were performed to determine how CD is associated with maternity, delivery and the neonatal period.

**Results:**

Regardless of sex, a reduction in CD risk was observed in children born to mothers aged ≥35 years (odds ratio [OR] 0.8; 95 % confidence interval [CI] 0.7–0.9) and with high maternal income (OR 0.9; 95 % CI 0.8–0.9). Being a second-born child, however, was positively associated with CD. Among boys, elective caesarean delivery increased the risk of CD (OR 1.2; 95 % CI 1.0–1.4), while maternal overweight (OR 0.9; 95 % CI 0.8-0.9), premature rupture of the membrane (OR 0.4; 95 % CI 0.2–0.8) and low birth weight showed a negative association. Girls had an increased CD risk compared to boys and in girls the risk was increased by repeated maternal urinary tract infections (OR 1.1; 95 % CI 1.0–1.2).

**Conclusions:**

Elective caesarean delivery and repeated maternal urinary tract infections during pregnancy are associated with increased risk of CD onset during childhood, suggesting the role of dysbiosis during early life. High maternal age and high income reduced the risk of CD, which might be due to infant-feeding practices and life style.

## Background

Celiac disease (CD) is an immune mediated enteropathy triggered by exposure to dietary gluten in genetically susceptible individuals [1]. Epidemiological studies suggest the role of environmental risk factors as exemplified by the worldwide recognition of CD, geographical variation in incidence and increase in CD frequency that cannot be fully explained by increased awareness among doctors and the public [2–6]. Sweden recorded pronounced differences in CD incidence between birth cohorts, best represented by the Swedish CD epidemic 1985–1996, but also by more recent variations [2]. Kondroshova et al. found a five-fold difference in CD prevalence between Finland and adjacent Karelia [7]. CD is almost twice as common in southern Sweden compared to the central and northern parts without any known genetic differences in the population [8]. However, the environmental factors that are of importance in explaining these differences are not yet fully established.

The immune system develops through interaction with the environmental factors and it is assumed that early life events, including fetal life, play a major role in this process. This includes interactions between the mother and the fetal immune system with tolerogenic processes that begin during fetal life [9]. Maternal health, including obesity, has been shown to influence immune development [10]. A well-functioning immune system is also dependent on a healthy gut microbiota. Colonization of the gut begins at delivery with the transferral of microflora from the maternal birth canal, faeces and skin. The neonates’ microbiota are thus affected by mode of delivery but also by factors affecting the maternal microbiota before delivery [11]. Changes in the infant’s microbiota have been shown to occur following birth, infections, antibiotic use and diet (in particular breastfeeding), thus influencing the immune system [12–14]. Given this background, factors related to maternal health and other early life events that impact these processes are of interest to study in relation to CD risk. Several previous studies have suggested that early life events related to maternity, pregnancy, delivery and neonatal life influence the risk of developing CD [15–18]. In the present study, we use a large database consisting of the entire Swedish child population and subsequent childhood CD cases identified nationwide with the aim of investigating how conditions related to maternity, delivery and the neonatal period influence the risk of getting a CD diagnosis during childhood.

## Methods

The study population consisted of 1 912 204 live births. The Umeå SIMSAM Lab (SIMSAM: Swedish Initiative for Research on Microdata in the Medical and Social Sciences) was used to access linked data on all children born in Sweden from 1991 to 2009 and their respective mothers. Data on the total population and perinatal events were obtained from the Medical Birth Register, income data were obtained from the Longitudinal Integration Database for Health Insurance and Labour Market Studies, and data on CD from the Swedish National Childhood CD Incidence Register [4]. Data linkage was performed by Statistics Sweden using the children’s Swedish personal identity number (PIN) [19].

### Celiac disease case ascertainment

CD ascertainment was based on the European Society for Paediatric Gastroenterology, Hepatology and Nutrition’s (ESPGHAN) diagnostic criteria from 1990 requiring villous atrophy on a normal diet followed by clinical remission on a gluten-free diet [20]. Small intestinal biopsies were first assessed according to the Alexander classification and thereafter by the Marsh-Oberhuber classification [21]. Other details concerning the register have been published earlier [4]. The study included 6 596 children who fulfilled the CD diagnostic criteria and were reported with a PIN necessary for data linkage.

### Exclusion criteria

Before data linkage, Statistics Sweden excluded all abortions, stillbirths, early neonatal deaths (≤30 days of birth) and new-borns whose birth weight was recorded as <1000 g. Also 1 498 (18.5 %) children were excluded because they were reported to the Swedish National Childhood CD Incidence Register without a PIN. To avoid misclassification of CD cases, we only included cases where villous atrophy had been confirmed according to ESPGHAN’s guidelines, see the previous section. We thus excluded cases without villous atrophy even if they had elevated serological markers and cases with minor enteropathy even if they had symptoms suggesting CD.

### Assessment of the maternity, pregnancy, delivery and neonatal life characteristics

We included 15 characteristics because either previous research suggested them as plausible in CD etiology and/or because of their potential relationship with the immune system development during childhood. Factors with possible effects on maternal health and life style included maternal age at delivery. This was categorized into four groups: <25, 25–29, 30–34 and ≥35 years (no information available for 0.1 %). Maternal disposable income during pregnancy with the index child (per 100 SEK) was categorized into three predefined strata based on quartile ranges (no information available for 1.3 % children). Maternal smoking during pregnancy was categorized into no smoking, smoking 1–9 cigarettes per day and smoking ≥10 cigarettes per day. Because of the absence of a linear relationship, we re-categorized this into yes if a mother smoked and no if no smoking was reported (Information was missing in 5.5 % children). Maternal body mass index (BMI) measured at first antenatal visit was divided into underweight (<18.5), normal weight (18.5–24.9), overweight (25.0–29.9) and obesity (≥30.0), (information was missing in 19.9 %).

As marker for exposure to infections the following variables were included; parity, maternal and neonatal infections. Parity was included as a measure of number of sibling, because number of siblings is associated with frequency of infections [22]. A child was defined as parity 1 if the mother had never given birth previously, parity 2 if the current child was the second child and parity ≥3 represented child number three and above (information was missing in 0.001%). Maternal infections during pregnancy and neonatal infections were included because they are associated with increased stress to the foetus. Additionally, children with infections are often treated with antibiotics that have an effect on the microbiota. These were classified according to the WHO International Classification of Diseases (ICD) 9th and/or 10th revisions (Table 1). Children were coded yes if the condition was reported and the rest were coded no. No missing information was reported.

**Table 1:** ICD codes used to identify diagnoses in this study

Description	ICD 10	ICD 9
Maternal infections		
Infections of genitourinary tract in pregnancy	O23	646.60, 646.61, 646.62, 646.63
Infections of amniotic sac and membranes	O41.1	658.41, 658.43
Pyrexia during labour, not elsewhere classified	O75.2	659.21
Other infection during labour	O75.3	659.31
Premature rupture of the membrane		
Premature rupture of the membrane, onset of labour	O42	
Neonatal infections		
Pneumonia	P23	770.0
Sepsis	P36	771.81
Other neonatal infections	P39	771.5, 771.6, 771.82, 771.89

*ICD* International Classification of Diseases

The Swedish ICD system used was constructed based on the ICD classification of the World Health Organization

Additional markers of health and stress of the foetus/child were as follows; duration of pregnancy, which was categorized as either born at gestational age <37 weeks or ≥37 weeks, (information was missing in 0.1% children) [23]. Infant birth weight in grams was categorized into very low birth weight (<1500 g), low birth weight (1500–2499 g) and normal birth weight (≥2500 g) (information on birth weight was missing in 0.3 %). Sex was either male or female and no data was missing. Small for gestational age was categorized into yes and no and data was missing in 3.3 % of children. Apgar score at five minutes after birth was recorded on a scale of 0–10: it was categorized into low Apgar score (<7) and normal Apgar score (≥7) (information was missing in 0.3 % children).

To study possible effects of delivery on gut microbiota, mode of delivery was categorized into caesarean and vaginal, caesarean delivery was further divided into elective and emergency. Premature rupture of the membrane (PROM) was classified according ICD 9th and/or 10th revisions.

### Statistical analysis

All statistical analyses were performed using SPSS 22 for Windows. For descriptive analysis, two-by-two tables were used to compare CD cases with non-cases. Bivariate analyses using logistic regression were performed to determine the independent associations between CD and the studied explanatory variables.

Multivariate logistic regression analyses were performed in order to adjust for multiple explanatory variables and thereby reduce any potential bias resulting from differences in the compared groups. The multivariate logistic regression models only included variables that were statistically significant in their preceding bivariate models. For categorical variables, all categories were retained as long as there was a category that was statistically significant in the bivariate analysis. All analyses were repeated separately for boys and girls, except in the post hoc analysis. Odds ratios (ORs) with 95 % confidence intervals (CIs) were calculated to estimate the risk of acquiring CD in the context of various conditions. Statistical significance was attained with a *p <*0.05, Missing data was excluded only for terms included in the analyses.

### Post hoc analyses

To determine if the nature of maternal infections during pregnancy influenced CD risk differently, we categorized infections into urinary tract infections and other infections and examined the risk of CD depending on these categories. We also hypothesized that CD risk may differ based on a combined effect of maternal age, income and urinary tract infections. Two groups were formed: mothers aged >35 years with high income and no urinary tract infections and these were compared with mothers aged <35 years, with middle/low income and with urinary tract infections.

## Results

### Background information

CD was almost twice as common in girls compared to boys. The highest proportion of mothers with children diagnosed with CD were aged 25–29 years and belonged to the middle-income quartile. In the study population, 15 % of the children were delivered by caesarean section. All basic characteristics of this population are shown in Table 2.

**Table 2:** Maternal and perinatal characteristics for children, with and without celiac disease (CD) born in Sweden during 1991–2009

Descriptives		CD Cases N (%) (Total = 6596)	Not CD cases N (%) (Total = 1 912 204)
Maternal age (years)	<25	1 155 (17.5)	329 634 (17.3)
	25–29	2 527 (38.3)	639 023 (33.6)
	30–34	2 077 (31.5)	612 299 (32.2)
	≥35	837 (12.7)	322 300 (16.9)
Disposable income (Per 100 SEK)	Low	2 224 (33.8)	627 366 (33.4)
	Middle	2 558 (38.9)	625 586 (33.4)
	High	1 799 (27.3)	627 253 (33.4)
Smoking during pregnancy	No	5 423 (86.4)	1 560 509 (86.6)
	Yes	853 (13.6)	240 677 (13.4)
Body mass index (kg/m^2^)	<18.5	148 (2.8)	40 844 (2.7)
	18.5–24.9	3 494 (65.8)	977 145 (64.1)
	25.0–29.9	1 190 (22.4)	358 566 (23.5)
	>30	481 (9.1)	147 426 (9.7)
Parity (n)	1	2 692 (40.8)	806 990 (42.3)
	2	2 657 (40.3)	695 173 (36.5)
	≥3	1 247 (18.9)	403 408 (21.2)
Duration of pregnancy (weeks)	37–45	6 256 (94.9)	1 788 057 (93.9)
	<37	335 (5.1)	115 524 (6.1)
Premature rupture of the membrane	No	6 546 (99.2)	1 883 116 (98.8)
	Yes	50 (0.8)	22 482 (1.2)
Mode of delivery	Vaginal	5 659 (85.8)	1 602 943 (84.9)
	Caesarean	937 (14.2)	284 634 (15.1)
Caesarean delivery	Elective	434 (46.3)	134 351 (47.2)
	Emergency	503 (53.7)	150 283 (52.8)
Infant birth weight (grams)	<1500	30 (0.5)	14 049 (0.7)
	1500–2499	212 (3.2)	67 129 (3.5)
	≥2500	6 336 (96.3)	1 818 885 (95.4)
Sex	Male	2 412 (36.6)	980 289 (51.4)
	Female	4 184 (63.4)	925 309 (48.6)
Small for gestational age	No	6 225 (97.8)	1 800454 (97.7)
	Yes	140 (2.2)	43 148 (2.3)
Apgar score at 5 min	≥7	6 513 (99.4)	1 869 284 (98.9)
	<7	42 (0.6)	20 894 (1.1)
Neonatal infections	No	6 521 (98.9)	1 885 798 (98.9)
	Yes	75 (1.14)	19 800 (1.0)
Maternal infections^a^	No	5 670 (86.0)	1 657 790 (87.0)
	Yes	926 (14.0)	247 808 (13.0)

*SEK* Swedish Krona

^a^Maternal infections; urinary tract infection and other maternal infections

### Bivariate and multivariate results for all children

In the bivariate analysis, increased childhood CD risk was significantly associated with maternal age 25–29 years (compared to <25 years), middle income (compared to low), being a second born child (compared to being the first), maternal infections in general during pregnancy (compared to no infections during pregnancy), and being a female (Table 3). Reduced CD risk was independently associated with maternal age ≥35 years (compared to <25 years), high maternal income (compared to low), maternal overweight (compared to normal weight), parity ≥3 (compared to being a first child), preterm delivery (compared to full term), PROM (compared to not PROM), very low birth weight (compared to normal), and low Apgar score at 5 min after delivery (compared to high). No independent association was found between CD and maternal smoking, maternal underweight or obesity, mode of delivery, low birth weight, being small for gestation age, neonatal infections and other maternal infections.

**Table 3:** Celiac disease risk in relation to each specific maternal and perinatal condition for all children and divided for boys and girls, respectively (Results from the bivariate analysis)

Maternal and perinatal exposures		All children		Boys		Girls	
		OR (95 % CI)	*P*-value	OR (95 % CI)	*P*-value	OR (9 5% CI)	*P*-value
Maternal age (years)	<25	1.0		1.0		1.0	
	25–29	1.1 (1.1–1.2)	0.01	1.1 (0.9–1.3)	0.06	1.1 (1.1–1.2)	0.005
	30–34	0.9 (0.9–1.0)	0.4	0.9 (0.9–1.1)	0.68	1.9 (1.9–1.1)	0.4
	≥35	0.7 (0.7–0.8)	<0.001	0.7 (0.6–0.9)	<0.001	0.8 (0.7–0.8)	<0.001
Disposable income (per 100 SEK)	Low	1.0		1.0		1.0	
	Middle	1.2 (1.1–1.2)	<0.001	1.1 (1.0–1.3)	0.07	1.2 (1.1–1.2)	<0.001
	High	0.8 (0.8–0.9)	<0.001	0.8 (0.7–0.9)	<0.001	0.8 (0.8–0.9)	<0.001
Smoking during pregnancy	No	1.0		1.0		1.0	
	Yes	1.0 (0.9–1.1)	0.59	0.9 (0.9–1.1)	0.57	1.1 (0.9–1.2)	0.25
Body mass index (kg/m^2^)	18.50–24.99	1.0		1.0		1.0	
	<18.50	1.0 (0.9–1.2)	0.87	0.9 (0.8–1.3)	0.95	1.0 (0.8–1.3)	0.86
	25.00–29.99	0.9 (0.9–0.9)	0.03	0.9 (0.8–0.9)	0.03	0.9 (0.9–1.0)	0.27
	≥30	0.9 (0.8–1.0)	0.06	0.9 (0.7–1.0)	0.05	0.9 (0.8–1.1)	0.40
Parity (n)	1	1.0		1.0		1.0	
	2	1.1 (1.1–1.2)	<0.001	1.1 (1.1–1.2)	0.01	1.2 (1.1–1.2)	<0.001
	≥3	0.9 (0.9–0.9)	0.03	0.9 (0.9–1.1)	0.51	0.9 (0.8–0.9)	0.02
Duration of pregnancy (weeks)	37–45	1.0		1.0		1.0	
	<37	0.8 (0.7–0.9)	0.001	0.8 (0.7–0.9)	0.04	0.9 (0.7–0.9)	0.02
Premature rupture of the membrane	No	1.00		1.0		1.00	
	Yes	0.6 (0.5–0.9)	0.002	0.4 (0.2–0.7)	0.001	0.8 (0.6–1.2)	0.28
Mode of delivery	Vaginal	1.0		1.0		1.0	
	Caesarean	0.9 (0.9–1.0)	0.09	0.9 (0.9–1.1)	0.49	0.9 (0.9–1.1)	0.25
Caesarean delivery	Vaginal	1.0		1.0		1.0	
	Elective	0.9 (0.8–1.0)	0.14	0.8 (0.7–1.0)	0.03	0.9 (0.9–1.1)	0.73
	Emergency	0.9 (0.9–1.1)	0.43	1.1 (0.9–1.2)	0.29	0.9 (0.8–1.1)	0.23
Infant birth weight (grams)	≥2500	1.0		1.0		1.0	
	1500–2499	0.9 (0.8–1.0)	0.16	0.9 (0.7–1.1)	0.38	0.9 (0.8–1.0)	0.15
	<1500	0.6 (0.4–0.9)	0.01	0.4 (0.2–0.8)	0.01	0.7 (0.5–1.1)	0.15
Sex	Male	1.0					
	Female	1.8 (1.8–1.9)	<0.001				
Small for gestational age	No	1.0		1.0		1.0	
	Yes	0.9 (0.8–1.1)	0.46	0.9 (0.7–1.3)	0.69	0.9 (0.8–1.2)	0.49
Apgar score at 5 min	≥7	1.0		1.0		1.0	
	<7	0.6 (0.4–0.8)	<0.001	0.8 (0.5–1.1)	0.18	0.5 (0.3–0.8)	0.001
Maternal infections^a^	No	1.0		1.0		1.0	
	Yes	1.1 (1.0–1.2)	0.01	1.0 (0.9–1.2)	0.67	1.1 (1.0–1.2)	0.005
Neonatal infections	No	1.0		1.0		1.0	
	Yes	1.1 (0.9–1.2)	0.43	1.2 (0.9–1.7)	0.24	1.1 (0.8–1.5)	0.50

*OR* odds ratio, *CI* confidence interval, *SEK* Swedish Krona: Bivariate logistic regression analyses were performed for all variables, and results reported regardless of statistical significance

^a^Includes both urinary tract infection and other maternal infections

In the multivariate analyses, increased CD risk remained statistically associated with maternal age 25–29 years, middle disposable income, being a second born child, being female, and with maternal infections (Table 3). A reduction in CD risk remained associated with maternal age ≥ 35 years, high disposable income, maternal overweight, PROM, and low Apgar score. CD was not associated with maternal underweight or obesity, parity ≥3, preterm birth and birth weight (Table 3).

### Sex specific bivariate and multivariate analyses

We found several factors that only had a relation to CD in one of the sexes. Among boys, elective caesarean delivery was associated with increased CD risk in the multivariate model despite an independent reduced CD risk (Table 4). In both the bivariate and the multivariate model, maternal overweight, PROM and very low birth weight were associated with reduced CD risk. Although low birth weight was associated with reduced CD risk in the bivariate model, this association was not statistically significant after adjustment. Specifically for girls, the bivariate and multivariate analyses showed increased CD risk to be associated with maternal infections while low Apgar score was associated with reduced CD risk. The effect of other factors remained similar in boys and girls except for marginal differences in the ORs.

**Table 4:** Celiac disease risk in relation to each specific maternal and perinatal condition for all children and divided for boys and girls, respectively (Results from the multivariate analysis)

Maternal and perinatal exposures		All children		Boys		Girls	
		OR (95 % CI)	*P*-value	OR (95 % CI)	*P*-value	OR (95 % CI)	*P*-value
Maternal age	<25	1.0		1.0		1.0	
	25–29	1.1 (1.0–1.2)	0.01	1.1 (1.0–1.3)	0.07	1.1 (1.0–1.2)	0.04
	30–34	1.0 (0.9–1.1)	0.75	1.0 (0.9–1.2)	0.87	1.0 (0.9–1.1)	0.52
	≥35	0.8 (0.7–0.9)	<0.001	0.8 (0.7–0.9)	0.01	0.8 (0.7–0.9)	<0.001
Disposable income (per 100 SEK)	Low	1.0		1.0		1.0	
	Middle	1.2 (1.1–1.2)	<0.001	1.1 (1.0–1.3)	0.01	1.2 (1.1–1.3)	<0.001
	High	0.9 (0.8–0.9)	<0.001	0.8 (0.7–0.9)	0.01	0.9 (0.8–0.9)	0.002
Body mass index (kg/m^2^)	18.50–24.99	1.0		1.00			
	<18.50	1.0 (0.8–1.2)	0.85	1.0 (0.7–1.3)	0.90		
	25.00–29.99	0.9 (0.9–0.9)	0.04	0.9 (0.8–0.9)	0.03		
	≥30	0.9 (0.8–1.0)	0.09	0.8 (0.7–0.9)	0.04		
Parity (n)	1	1.0		1.00		1.00	
	2	1.2 (1.1–1.2)	<0.001	1.2 (1.0–1.3)	0.01	1.2 (1.1–1.2)	<0.001
	≥3	1.0 (0.9–1.1)	0.58	1.1 (0.9–1.2)	0.34	1.0 (0.9–1.1)	0.53
Duration of pregnancy (weeks)	37–45	1.0		1.00		1.00	
	<37	0.9 (0.8–1.2)	0.86	1.1 (0.8–1.4)	0.62	0.9 (0.8–1.0)	0.90
Premature rupture of the membrane	No	1.00		1.00			
	Yes	0.7 (0.5–0.9)	0.02	0.4 (0.2–0.8)	0.01		
Mode of delivery	Vaginal			1.00			
	Elective			1.2 (1.0–1.4)	0.02		
Infant birth weight (grams)	≥2500	1.0		1.00			
	1500–2499	1.0 (0.9–1.2)	0.73	1.0 (0.7–1.3)	0.97		
	<1500	0.8 (0.5–1.2)	0.27	0.3 (0.1–0.9)	0.03		
Sex	Male	1.0					
	Female	1.8 (1.7–1.9)	<0.001				
Apgar score at 5 min	≥7	1.0				1.00	
	<7	0.7 (0.5–0.9)	0.02			0.5 (0.3–0.8)	0.003
Maternal infections^a^	No	1.0				1.00	
	Yes	1.1 (1.1–1.2)	<0.001			1.1 (1.0–1.2)	0.006

*OR* odds ratio, *CI* confidence interval, *SEK* Swedish Krona

Multivariate logistic regression including all variables found to be statistically significant (*p <*0.05) in the bivariate analyses (Table 3)

^a^Includes both urinary tract infections and other maternal infections

### Post hoc analyses

Both bivariate and multivariate results showed increased CD risk to be associated with maternal urinary tract infections, while other maternal infections showed no association (Table 5). In further stratification, CD risk was significantly reduced in children with mothers who had no urinary tract infections, were aged ≥35 years and had high income.

**Table 5:** Celiac disease risk in relation to maternal infections during early life in all children

	Descriptive					
Exposures	CD Cases	Not CD cases	Bivariate analyses		Multivariable analyses	
	*N* (%)	*N* (%)	OR (95 % CI)	*P*-value	OR (95 % CI)	*P*-value
Urinary tract infections						
No	5 700 (86)	1 667 663 (88)	1.0		1.0	
Yes	896 (14)	237 935 (13)	1.1 (1.0–1.2)	0.007	1.2 (1.1–1.2)^a^	<0.001
Other maternal infections						
No	6 561 (99)	1 893 642 (99)	1.0		1.0	
Yes	35 (0.5)	11 956 (0.6)	0.8 (0.6–1.2)	0.32	0.9 (0.7–1.4)^a^	0.80
urinary tract infections, <35years, Low income						
Yes	689 (63)	136 479 (48)	1.0		1.0	
No	343 (37)	150 895 (53)	0.5 (0.5–0.6)	<0.001	0.5 (0.4–0.6)^b^	<0.001

^a^Adjusted for maternal age, disposable income, body mass index, parity, and duration of pregnancy, infant birth weight, sex, and Apgar score and urinary tract infections

^b^Adjusted for body mass index, parity, pregnancy duration, and premature rupture of the membrane, infant birth weight, sex, Apgar score and other maternal infections

## Discussion

Our results showed that the risk of developing CD during childhood was associated with several environmental exposures during pregnancy and neonatal period. A reduced risk was associated with high maternal age and high maternal disposable income, while being a second born child was associated with increased risk. Specifically for boys, increased CD risk was associated with elective caesarean delivery, but negatively associated with maternal overweight, premature rupture of the membrane and low birth weight. Among girls, CD risk was positively associated with maternal infections, but negatively associated with low Apgar score at 5 min. We also found that combining several factors that seemed to have a strong effect (high maternal age, high income and no repeated urinary tract infections had a synergistic effect of reducing CD risk.

One of the strength of this study is that it was based on the entire child population in Sweden born during between 1991 and 2009 and ~6 500 biopsy-verified CD cases, which provided enough statistical power to guarantee high precision in our estimates and enabled adjustment for several variables. The high number of CD cases also enabled sub-analyses by age group, comparing children diagnosed at age <2 years and 2–14.9 years, respectively, although no major differences were found (data not shown).

Another strength, our CD cases were identified through the Swedish National Childhood CD Incidence Register reported from all paediatric departments across the country [4]. We used strict diagnostic criteria to avoid misclassification. It is important to note that during this study period there were no changes in the national recommendations on paediatric CD diagnosis, moreover the revised ESPGHAN guidelines were introduced later, in 2012 [24].

One of the limitations of this study was that some potentially interesting information was missing, which is a consequence of relying on data from registers. As shown, the proportion of individuals with missing information on the studied exposures was ~0–20 %. However, this did not differ between CD cases and non-cases. Moreover, data on risk factors was prospectively collected and this was done separately prior to linkage with CD data. This is an advantage since it eliminates the problem of recall bias that often affects retrospective studies. Another limitation is that CD cases without a PIN code were excluded. The main reason why some lacked a PIN code was because some parents were not asked for informed consent, while a few declined to participate. It can be speculated that parents with lower income more often than others declined to participate; however, this is unlikely and it could not have influenced our results in any major way.

In this study the association between maternal age and CD risk indicated no progression in risk and it is not obvious why different maternal ages would influence CD risk differently. We hypothesize that maternal age at delivery might be a marker for lifestyle aspects for example older mothers are more likely to practise prolonged and exclusive breastfeeding [25–27]. The gut microbiota of breastfed infants differ in composition compared to formula fed infants [11]. Moreover, human milk contains immunoactive substances and present food antigens in small proportions that might have a positive effect on the tolerogenic process [14]. Earlier studies suggested a protective effect of breastfeeding against CD and are summarized in a meta-analysis published by Akobeng and Thomas [28]. However, more recent studies could not confirm this association [29–32]. Worth noting is that all studies on breastfeeding and CD are observational in nature. Performing randomized studies is impossible for ethical reasons since breastfeeding is considered superior to formula feeding. Unfortunately, we lacked data on breastfeeding and could not show a direct association. We also considered parity and disposable income in the adjusted model but only found marginal effect and without proven interaction.

Lately, gut microbiota has received much attention in relation the development of immunological diseases, CD included. Our living conditions have changed substantially with effects on diet, microbiota and infectious pattern, including antibiotic use. These changes influence our immune system, increasing the risk of allergic and autoimmune diseases. Against this background, the well-known hygiene hypothesis was first suggested by Strachan in 1989 [33]. Several studies have been made in developed countries that indicate an increase in CD [3, 34]. Kondroshova et al. demonstrated that the hygiene hypothesis is perusable in CD development [7].

In line with the hygiene hypothesis, disturbed microbiota, or dysbiosis, have received more and more attention in CD research [35]. In the present study, we found an increased risk of CD in boys born with elective caesarean section. Similar results have been shown previously but without sex related differences [17]. Studies on Crohn’s disease have also identified elective caesarean delivery as a risk factor [36, 37]. Children born by elective caesarean delivery are not exposed to the vaginal or faecal microbiota in contrast with children born through emergency caesarean delivery after initiated labour and ruptured membranes that get exposed to vaginal microbiota. We also found increased CD risk to be associated with maternal infections during pregnancy, in particular repeated urinary tract infections. During pregnancy, antibiotics are used to treat urinary tract infections, which could affect the gut microbiota that is later transferred to the neonate. Additionally, antibiotics have also been shown to increase CD risk directly [38]. Both elective caesarean section and repeated antibiotic treatment for urinary tract infections may lead to dysbiosis, resulting in disturbed maturation of the immune system in the child. Gut microbiota affect gut permeability, gut inflammatory activity (both directly and via the release of metabolites) and dysbiosis, all of which are suspected to play a role of increasing the risk of autoimmune disorders [39]. Moreover, we found a rather large reduction in CD risk to be associated with PROM. Due to the ruptured membranes, the baby is exposed to the maternal vaginal microbiota that is rich in lactobacillus species [40] for longer duration compared to normal delivery, theoretically this results in early colonization with lactobacilli. Lactobacilli have been shown to have a modulating effect on the immune response to gluten in cell culture and mouse models and could contribute to the protective effect of PROM seen in this study [41, 42]. However, this finding should be interpreted with caution since there were few cases and this hypothesis is speculative.

We found high income to be associated with reduced risk of CD during childhood while middle income was associated with increased risk. Previous studies both support and contradict our finding [16, 43–46]. Income could be associated with health during pregnancy, breastfeeding and early infant feeding. It is also possible that the effect of income is not associated with the child’s foetal and early life, but with life style factors whose effect begin during later childhood, for example the amount of gluten ingested or other dietary factors. Possibly, health care seeking behaviour is dependent on income status. However, in Sweden health care is free up to the age of 18 and this should thus reduce this effect. Current evidence also suggests that individuals from low socio-economic positions are more likely to refrain from seeking health care, which would imply an underestimation of the effect [47]. Information on education could have offered better clarity but unfortunately was not available. We further compared children with mothers aged ≥35 years, with high income and no urinary tract infection versus the other mothers and found synergistic effects on CD risk. We also report a positive but not statistically significant association between neonatal infections and CD, although earlier studies have shown a significant positive association [48, 49].

We observed a protective effect due to maternal overweight and low birth weight in boys and low Apgar score in girls. High parity was also related to reduced CD risk, this did not support the hypothesis suggesting high parity to increase the infectious load of the index child. In a previous case-referent study, number of siblings did not affect CD risk [49]. Maternal overweight and low birth weight were associated with reduced CD risk. These findings were unexpected since both factors are known to have a negative impact on health. It is possible that these were chance findings or were due to residual confounding therefore interpretation should be done with caution.

Several variables were included and were tested for collinearity but found no such evidence. We conclude that the shown effects most likely represent the true magnitude in the adjusted models. In this study, the most significant findings had an OR close to 1. This means that their impact on CD risk could be regarded as low to moderate. CD is a multifactorial disease with HLA DQ2 or HLA DQ8 and gluten as necessary causes. In addition to this, there are probably several environmental factors (some of which have been identified and others yet to be identified) that could contribute to CD risk. Thus it was unlikely that this study could identify variables with higher than the observed explanatory value for the risk of being diagnosed with CD.

## Conclusions

In this study, we found indications that elective caesarean section and maternal urinary tract infections are associated with increased risk of being diagnosed with CD during childhood; these two factors are related to possible gut dysbiosis. Additionally both findings were sex specific; differences in CD risk between boys and girls have been reported before and justify separate analysis in CD studies [44]. As a matter of fact, the considerable and well known higher CD risk in girls seen long before puberty is an interesting phenomenon that should be further studied [50]. We also found that maternal age with possible association to life style and diet (in particular breast feeding) is inversely related to CD risk. All factors could be linked to the development of the immune system. In future, preventive strategies could benefit by taking measures to prevent gut dysbiosis. Reduced prescription of antibiotics is one way that would also work to counteract the enormous problem with development of bacterial resistance to antibiotics. Promoting normal delivery would also be in line with goals in the field of maternal and child health. For some of our findings for example, increased CD risk was associated with being female, having maternal age 25–29 and being a second born, we could not establish a pathological mechanism. However it is worth remembering that one important role of epidemiological studies is to generate new hypotheses and thereby contribute to future search for disease mechanisms, possible treatment and prevention.

## Abbreviations

BMI, body mass index; CD, celiac disease; CI, confidence intervals; OR, odds ratio; PIN, personal identification number; PROM, premature rupture of the membrane
